# An updated management approach of Pompe disease patients with high-sustained anti-rhGAA IgG antibody titers: experience with bortezomib-based immunomodulation

**DOI:** 10.3389/fimmu.2024.1360369

**Published:** 2024-03-08

**Authors:** Ankit K. Desai, Garima Shrivastava, Christina L. Grant, Raymond Y. Wang, Trevor D. Burt, Priya S. Kishnani

**Affiliations:** ^1^ Division of Medical Genetics, Department of Pediatrics, Duke University Medical Center, Durham, NC, United States; ^2^ Division of Genetics and Metabolism, Children’s National Hospital, Washington, DC, United States; ^3^ Division of Metabolic Disorders, Children’s Hospital of Orange County, Orange, CA, United States; ^4^ Department of Pediatrics, University of California-Irvine School of Medicine, Orange, CA, United States; ^5^ Division of Neonatology, Department of Pediatrics, Duke University School of Medicine, Durham, NC, United States; ^6^ Children’s Health and Discovery Initiative, Duke University School of Medicine, Durham, NC, United States

**Keywords:** alglucosidase alfa, anti-drug antibodies, entrenched immune tolerance induction, bortezomib, Pompe disease, anti-rhGAA IgG antibodies, enzyme replacement therapy

## Abstract

**Introduction:**

High sustained anti-rhGAA antibody titers (HSAT; ≥12,800) are directly linked to reduced efficacy of enzyme replacement therapy (ERT) and subsequent clinical deterioration in infantile-onset Pompe disease (IOPD). We have previously demonstrated the safety and effectiveness of a bortezomib-based immune-tolerance induction (ITI) regimen (bortezomib, rituximab, methotrexate, and IVIG) in eliminating HSAT.

**Methods:**

Here, we describe two IOPD cases (patients 6 and 8) who developed HSAT at 8 and 10 weeks on ERT despite transient low-dose methotrexate ITI administration in the ERT-naïve setting and were treated with a bortezomib-based ITI regimen, and we compare their courses to a series of six historical patients (patients 1-5, and 7) with a similar presentation who exemplify our evolving approach to treatment.

**Results:**

In total, patients 6 and 8 received 16 and 8 doses of bortezomib (4 doses=1 cycle) respectively reducing titers from 25,600 to seronegative, but differences in the course of their therapy were instructive regarding the optimal approach to initial treatment of HSAT; specifically, patient 6 was treated initially with only a single course of bortezomib rescue therapy, while patient 8 received two back-to-back courses. Patient 8 received IVIG therapy throughout the immunosuppression whereas patient 6 received IVIG therapy and was switched to subcutaneous IgG replacement. Patient 6 had a transient reduction in anti-rhGAA antibodies, after receiving a single initial cycle of bortezomib, but had a recurrence of high anti-rhGAA antibody titer after 160 weeks that required 3 additional cycles of bortezomib to ultimately achieve tolerance. In contrast, patient 8 achieved tolerance after being given two consecutive cycles of bortezomib during their initial treatment and had B cell recovery by week 54. Since the reduction in anti-rhGAA antibodies, both patients are doing well clinically, and have decreasing ALT, AST, and CK. No major infections leading to interruption of treatment were observed in either patient. The bortezomib-based ITI was safe and well-tolerated, and patients continue to receive ERT at 40 mg/kg/week.

**Discussion:**

These case studies and our previous experience suggest that to achieve an effective reduction of anti-rhGAA antibodies in the setting of HSAT, bortezomib should be initiated at the earliest sign of high anti-rhGAA antibodies with a minimum of two consecutive cycles as shown in the case of patient 8. It is important to note that, despite initiation of ERT at age 2.3 weeks, patient 8 quickly developed HSAT. We recommend close monitoring of anti-rhGAA antibodies and early intervention with ITI as soon as significantly elevated anti-rhGAA antibody titers are noted.

## 1 Introduction

Pompe disease (OMIM no. 232300, glycogen storage disease type II) is an autosomal recessive neuromuscular disorder caused by pathogenic variants in the *GAA* gene (OMIM no. 606800) encoding acid alpha-glucosidase (GAA) enzyme. Deficiency of GAA results in pathological glycogen accumulation in the lysosomes of multiple tissues, especially cardiac, skeletal, and smooth muscles (1). Infantile-onset Pompe disease (IOPD) presents in the first few days to weeks of life (1). It is characterized by progressive muscle weakness, hypertrophic cardiomyopathy, respiratory distress, hypotonia, and if left untreated, death within two years of life due to cardiorespiratory failure (1, 2).

Enzyme replacement therapy (ERT) with recombinant human acid alpha-glucosidase (rhGAA) has significantly improved overall, and ventilator-free survival, and resulted in an improvement in motor milestones in many children with IOPD but has certain limitations (3, 4). The response to ERT has been heterogeneous and is affected by multiple factors including cross-reactive immunologic material (CRIM) status (i.e., presence or absence of any GAA expression), anti-rhGAA antibodies, age at diagnosis, dose of ERT (recommended dose – 20 mg/kg every other week), and extent of muscle damage at the time of treatment initiation (5). The development of high titer IgG antibodies against ERT can affect pharmacokinetics, and result in the need for invasive ventilation associated with disease progression despite high doses of ERT. It can also result in infusion-associated reactions (IARs) (6–8). The rapid elimination of cells that produce high titer anti-rhGAA IgG antibodies is very important in IOPD because disease progression is extremely rapid, and a delay in initiation of treatment by even a few days can impact the outcome (9–11).

The combination of rituximab, methotrexate, and IVIG has been shown to be most successful in inducing immune tolerance to ERT in high-risk, CRIM-negative IOPD patients when initiated in ERT-naïve settings (12). For patients who are CRIM-positive, who are typically considered to be at lower risk of developing anti-rhGAA antibodies, ITI with transient low-dose methotrexate (TLD-MTX) has been successful at inducing tolerance (13). Despite the progress made in immunomodulation approaches used in the ERT-naïve setting, some patients have a break in tolerance. The use of plasma cell targeting agents such as bortezomib (a proteasome inhibitor that affects both short-lived and long-lived plasma cells) and daratumumab (an anti-CD38 monoclonal antibody) in combination with rituximab, methotrexate, and IVIG has been successful in eliminating high-sustained anti-rhGAA antibody titers (HSAT) against ERT in patients with Pompe disease and other lysosomal storage diseases (LSDs) (14, 15). However, elimination of HSAT is a challenge, often requiring prolonged immune suppression to ensure long-term immune tolerance.

We originally reported on three Pompe disease patients who were successfully immune tolerized with a bortezomib-based immunomodulation regimen after experiencing clinical decline due to the development of HSAT (14, 16). In these 3 cases (**Table 1**; patients 1, 2, and 3), immunomodulation with a bortezomib-based regimen resulted in a significant reduction in antibody titers; patient 1 had a 2,048-fold (1:204,800 to 1:100) and patients 2 (1:409,600 to 1:6400) and 3 (1:204,600 to 1:3,200) had a 64-fold reduction in anti-rhGAA antibodies with no side-effects. The reduction in anti-rhGAA IgG antibody titers led to the improvement in overall clinical status; a decrease in left ventricular mass index (LVMI) with normalization of LVMI in two patients, a significant reduction in urinary glucose tetrasaccharide (Glc_4_), less dependence on mechanical ventilation and enteral feeding, and improvement in motor status. Despite the success of the protocol, the duration of immune suppression was necessarily long to overcome the entrenched anti-rhGAA immune response.

**Table 1:** Summary of age at diagnosis, CRIM status, age at ERT initiation, development of HSAT, and ITI with bortezomib in patients with IOPD and early-onset LOPD.

Patient ID	1*	2*	3*	4*	5*	6	7*	8
Treatment year	2010-2013	2010-2013	2010-2013	2010-2013	2018-2019	2018-2019	2020-2021	2020-2021
Publication	(Banugaria, Prater et al., 2013) (14)	(Banugaria, Prater et al., 2013) (14)	(Banugaria, Prater et al., 2013) (14)	(Stenger, Kazi et al., 2015) (17)	(Kim, Desai et al., 2023) (18)	N/A	(Curelaru, Desai et al., 2022) (19)	N/A
CRIM status	Positive	Negative	Positive	Positive	Positive	Positive	Negative	Positive
Phenotype	IOPD	IOPD	LOPD	IOPD	LOPD	IOPD	IOPD	IOPD
Age at Diagnosis	5.0 months	4.0 months	3.5 years	N/A	9 months	3.7 months	4.5 months	2.0 weeks
Age at ERT initiation	6.0 months	4.5 months	3.6 years	3.3 weeks	20 months	4.2 months	4.5 months	2.3 weeks
ITI in naïve setting	None	None	None	None	None	TLD-MTX	RTX+MTX+IVIG	TLD-MTX
Highest antibody titers (time since ERT initiation)	204,800 (Week 64 to 86)	819,200 (Week 86)	204,800 (Week 64 to 90)	204,800 (Week 42)	51,200 (Week 656)	25,600 (Week 21)	12800 (Week 24)	25,600 (Week 10)
Time on ERT at Bortezomib initiation	99 weeks	154 weeks	88 weeks	44 weeks	686 weeks	22 weeks	25 weeks	13 weeks
Time to initiate ITI since development of HSAT	51 weeks	111 weeks	32 weeks	22 weeks	38 weeks	1 week	1 week	3 weeks
Number of bortezomib cycles	3	4	6	2	2	4	2**	2**
Antibody titers at the study end (time since ERT initiation)	0 (383 weeks)	1,600 (423 weeks)	800 (354 weeks)	1,600 (82 weeks)	200 (851 weeks)	200 (278 weeks)	0 (109 weeks)	0 (93 weeks)

ITI, immune tolerance induction; CRIM, cross-reactive immunologic material; ERT, enzyme replacement therapy; HSAT, high and sustained anti-rhGAA IgG antibody titers; RTX, rituximab; MTX, methotrexate; TLD-MTX, transient low-dose methotrexate.

*Cases 1, 2, 3, 4, 5, and 7 have been previously published (14, 16–19).

**Two consecutive cycles of bortezomib were administered in patients 7 and 8.

To better understand antibody titer trends that could help us differentiate IOPD patients who are at risk for HSAT vs. low titers in the early phase of ERT treatment, potentially identifying patients who would benefit from earlier intervention, longitudinal anti-rhGAA antibody response in 37 CRIM-positive IOPD patients was analyzed (20). Of these 37 patients, 12 (32%) patients developed antibody titers of ≥12,800. In these patients who developed HSAT (n=12), the median time since ERT to first develop a titer of ≥12,800 was 10 weeks (range, 4-24 weeks). Twenty-five CRIM-positive IOPD patients who maintained antibody titers of <12,800 were classified as low titer patients. The median time to seroconversion in the low titer group was 8 weeks (range, 4-64 weeks) on ERT. The data suggested that the Pompe disease patients who will not tolerize to ERT are those who mount titers of ≥12,800 and an upward trend of antibody titers within the first 24 weeks on ERT. The data generated from this chart review identified different arcs in immune response to ERT after the initial exposure, which has allowed us to identify high-risk patients earlier in order to initiate bortezomib-based ITI.

In this article, we summarize the lessons learned from patients previously reported who received a bortezomib-based ITI regimen and report on two new CRIM-positive IOPD patients who developed high antibody titers (14, 16–19). Both newly reported patients failed to achieve durable immune tolerance despite routine immune modulation with TLD-MTX in the ERT-naïve setting. Both patients subsequently received rescue ITI with a bortezomib-based protocol at the earliest recognition of elevated titers. The number of bortezomib cycles at the time of initiation differed, however, which drastically changed the trajectory of the response. Through these cases and our previously published cases, we highlight the need for close monitoring of anti-rhGAA antibodies and an aggressive approach at the outset to allow for success in reducing anti-rhGAA antibodies.

## 2 Methods

### 2.1 Study design and patient inclusion criteria

A retrospective chart review on two CRIM-positive IOPD patients (patient 6 and 8) who received ITI with bortezomib, rituximab, methotrexate, and IVIG after developing HSAT was performed (16). The clinical data including *GAA* variants, CRIM status, age at ERT, anti-rhGAA antibody titer trends, and biomarkers were analyzed. Upon identification of a Pompe disease patient with HSAT, the detailed ITI protocol including the dosing schedule of immunomodulatory agents (bortezomib, rituximab, methotrexate, and IVIG) and safety monitoring was shared with the patient’s treating physician. Anti-rhGAA IgG antibody titers were determined by Sanofi Genzyme and/or LabCorp as previously described (36). Urinary Glc_4_ was determined as described previously (21, 22). All the relevant information was extracted from medical records provided by the principal care provider or physician of the respective patient. These patients’ medical records were examined and analyzed up until April 2023. The lessons from 37 CRIM-positive IOPD cases treated with ERT monotherapy previously published by our group helped in defining optimal timing for initiating a Pompe disease patient on a bortezomib-based ITI protocol (20). The endpoints such as age at diagnosis, age at ERT initiation, highest anti-rhGAA IgG antibody titer, time on ERT at bortezomib initiation, duration between development HSAT titer to bortezomib initiation, number of bortezomib cycles, and antibody titer at study end were compared to six previously published patients (patients 1, 2, 3, 4, 5, and 7) who were treated with the same ITI approach (14, 16, 17). Patients 1, 2, 4, and 7 had IOPD and patients 3 and 5 had late-onset Pompe disease (LOPD).

### 2.2 Study approval

Both newly reported patients were enrolled in a Duke institutional review board (IRB) approved study protocol (Pro00001562; Determination of Cross-Reactive Immunological Material [CRIM] Status) and/or Longitudinal Follow-up of Individual and Pompe disease (Pro100010830). A written informed consent was provided by the respective parents/guardians.

## 3 Case reports

Patient demographics, anti-rhGAA antibody titers, creatinine kinase (CK), urinary Glc4, and longitudinal immunoglobulin levels for both newly reported patients (patient 6 and 8) are shown in **Table 2**; **Supplementary Figure 1**.

**Table 2:** Patient demographics, treatment details, anti-rhGAA antibodies, and biomarkers.

	Patient 6	Patient 8
Gender	Female	Male
CRIM status	Positive	Positive
*GAA* variant 1 (Amino acid change)	c.799_803delins (p.Leu267Serfs*46)	c.1913G>C (p.Gly638Val)
*GAA* variant 2 (Amino acid change)	c.670C>T (p.Arg224Trp)	c.307T>G (p.Cys103Gly)
Age at ERT initiation	18.4 weeks	2.3 weeks
ITI in ERT-naïve setting	Methotrexate only	Methotrexate only
Age at Seroconversion (time since ERT initiation)	22.6 weeks (4.1 weeks)	6.3 weeks (4 weeks)
Peak anti-rhGAA antibody titers (time since ERT initiation)	25,600 (21 weeks)	25,600 (10 weeks)
Anti-rhGAA antibody titers at Bortezomib start (time since ERT initiation)	25,600 (21 weeks)	6,400 (13 weeks)
Most recent anti-rhGAA IgG titers (time since ERT initiation)	100 (278 weeks)	0 (93 weeks)
CK at ERT initiation (U/L)	759	529
CK at bortezomib start (U/L) (time since ERT initiation)	740 (25 weeks)	256 (10 weeks)
Most recent CK (U/L) (time since ERT initiation)	153 (286 weeks)	237 (83 weeks)
Urinary Glc_4_ at ERT initiation (mmol/mol creatinine)	45.8	8.9
Urinary Glc_4_ at Bortezomib start (mmol/mol creatinine) (time since ERT initiation)	15.1 (25 weeks)	5.9 (13 weeks)
Most recent Urinary Glc_4_ (mmol/mol creatinine) (time since ERT initiation)	33.4 (286 weeks)	4.6 (83 weeks)

ITI, immune tolerance induction; CRIM, cross-reactive immunologic material; ERT, enzyme replacement therapy; CK, creatine kinase; Glc_4_, glucose tetrasaccharide.

### 3.1 Patient 6

Patient 6 is a 6 years 9 months old Caucasian female who was clinically diagnosed with IOPD at the age of 3.7 months. At 2 days of life, the patient presented with a hypertrophic cardiomyopathy. At age 3 months, she had severe concentric left ventricular hypertrophy with mild subaortic flow acceleration from dynamic compression (LVM; 264.7 gm/m^2^), very poor feeding, and elevated CK levels (759 U/L). At age 3.7 months, the patient was diagnosed with CRIM-positive IOPD and ERT was initiated at 4.2 months of age with a dose of 20 mg/kg/week along with TLD-MTX ITI as previously published (**Table 2**) (13). Due to viral and Respiratory Syncytial Virus (RSV) infection, the 2^nd^ and 3^rd^ cycles of methotrexate were postponed and administered with 4^th^ and 5^th^ infusions instead of 2^nd^ and 3^rd^ ERT infusions. ERT dose was increased to 30 mg/kg/week at week 4 and was further increased to 40 mg/kg/week at week 7.

**Figure 1:**
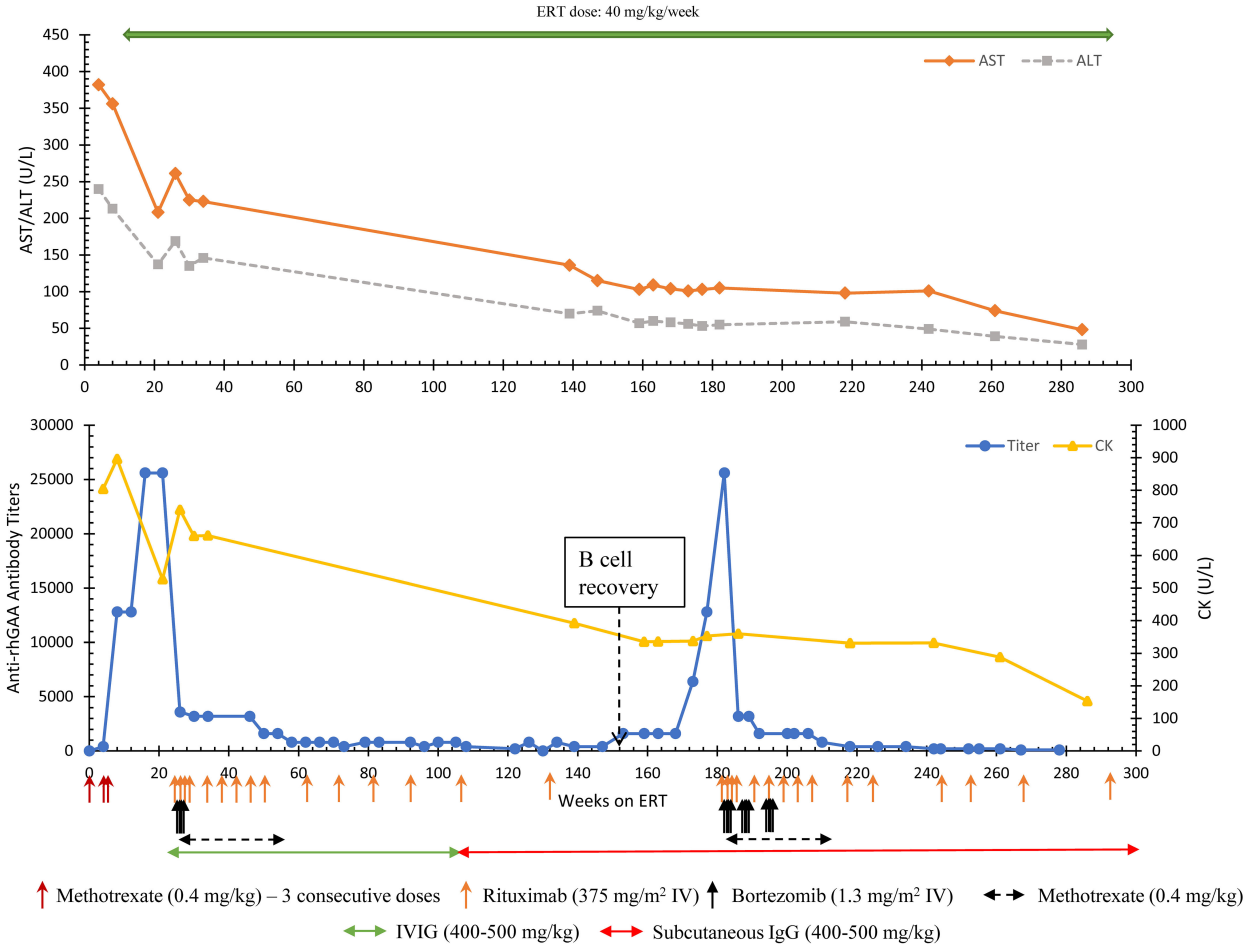
Longitudinal anti-rhGAA antibody titers, AST, ALT, and CK trends in Patient 6. AST, Aspartate aminotransferase; ALT, Alanine aminotransferase; CK, Creatine kinase; ERT, enzyme replacement therapy.

The patient seroconverted at week 4 (antibody titer 400) with increased antibody titers of 12,800 and 25,600 noted at weeks 8 and week 22, respectively. The increase in anti-rhGAA antibodies was associated with a plateau of gross developmental progress, marked oropharyngeal dysphagia, and high levels of CK (896 U/L), AST (356 U/L), ALT (213 U/L), and LVMI (144.19 gm/m^2^). At this point, the patient (age 9 months) received the first cycle of bortezomib-based immunomodulation at 22 weeks on ERT (14). After 4 doses of bortezomib (cycle 1), the patient received rituximab every 8 weeks for 6 months which was later stretched to every 12 weeks with monthly IVIG, and eventually discontinued with the last dose given week 134 (**Figure 1**). Methotrexate was spaced out and then was discontinued due to neutropenia and mouth sores that significantly impacted oral feeds. After receiving the initial cycle of ITI with bortezomib, anti-rhGAA antibody titers decreased from 25,600 (week 21) to 400 (week 73) and the patient continued to maintain low antibody titers in ranges of 200-800 until week 147. Urinary Glc_4_ decreased from 45.8 mmol/mol creatinine (week 1) to 13.2 mmol/mol creatinine (week 33). CK levels gradually decreased from 896 U/L (week 8) to 337 U/L (week 118) and remained low. At week 137, AST (136 U/L) and ALT (70 U/L) were improved, and the patient gained physical strength, could jump, run, and tumble sault achieved age-appropriate major motor milestones (week 120).

Following the first round of ITI, a complete recovery of B cells (normalization of CD19%) was observed at week 152. At that time, a steady rise in antibody titers was noted with antibody titers reaching 12,800 and 25,600 at weeks 177 and 182, respectively (**Figure 1**). An increase in the frequency of falls was noted. CK (353 U/L), AST (103 U/L), and ALT (53U/L) remained at her baseline. Considering the history and increasing titers, a 2nd round of bortezomib-based ITI with two consecutive cycles (8 doses) of bortezomib was initiated at week 182. An additional 4 doses of bortezomib were given at week 192 to avoid a further rise in titer. After 3 cycles of bortezomib, anti-rhGAA antibody titers decreased from 25,600 (week 182) to 800 (week 210), eventually becoming undetectable by week 286. The patient received maintenance rituximab, methotrexate, and subcutaneous IgG (due to a national shortage of IVIG during the COVID-19 pandemic) (**Figure 1**). The patient’s physical assessments in the period between recovery of B cells and developing high anti-rhGAA antibody titers were deferred due to COVID-19 restrictions and limited lab tests were done with the help of a home nurse. The patient did not lose physical milestones attained after the first round of bortezomib-based ITI but had an increase in dysphagia, speech delay, and hypernasal speech (week 179). No major infections leading to interruption in treatment were reported in the patient. However, there was an increase in the length of viral upper respiratory tract infection compared to healthy sibling while on IVIG that was normalized on Subcutaneous IgG and more stable IgG levels. At 331 weeks on ERT, chronic Haemophilus influenzae pneumonia was diagnosed based on bronchoscopy and was successfully treated with oral antibiotics.

Formal gross motor skills evaluations were performed at the initiation of the second round of ITI using Peabody Developmental Motor Scales. Stationary motor skills were measured at 33-month age equivalent, 9%ile for age, locomotion score was 52-month age equivalent, 75%ile for age (week 183, patient chronological age 46 months) with notable abnormal posture including increased lumbar lordosis, scapular winging, genu valgum, and pes planus. In-person, outpatient physical therapy was performed on hospital treatment days (her usual outpatient physical therapy had been entirely virtual due to the COVID pandemic). By week 209, stationary gross motor scores increased to 75%ile for age, and locomotive skills remained 75%ile for age. These scores have been maintained; at week 262 stationary score is 63%ile for age and locomotive score is 84%ile for age.

At the time of the report (week 267), the patient had significantly improved pulmonary condition with a few respiratory issues. Per her treating physician, she has minimal feeding difficulty, no significant cardiac involvement (normal LVMI; 45.4 gm/m^2^ at week 266), no significant motor skills deficit, and leads a functionally active lifestyle including riding a bicycle. A continued downward trend was noted in biomarkers with AST (28 U/L), ALT (48 U/L), and CK (153 U/L) remaining close to upper normal limits at week 286. The patient has not received any immunizations other than the annual flu shot due to ongoing immunosuppression and absence of B cells (week 282). At the last follow-up, the patient is receiving ERT infusions at 40 mg/kg/week, rituximab (every 16 weeks) and subcutaneous IgG (every 2 weeks) and is under observation for any break in tolerance; anti-rhGAA antibodies remain undetectable.

### 3.2 Patient 8

Patient 8 is a 2-year-10-month-old Caucasian male who was diagnosed via newborn screening (NBS) with CRIM-positive IOPD. Two pathogenic (c.1913G>T and c.307T>G) variants were identified. At age 2 weeks, the patient presented with ventricular hypertrophy, feeding difficulty, and hypotonia. Left ventricular mass index (LVMI) (112 g/m^2^), urinary Glc_4_ (27.6 mmol/mol creatinine), CK (483 U/L), AST (144 U/L), and ALT (80 U/L) were elevated. The patient was initiated on ERT at age 2.3 weeks with a dose of 15 mg/kg weekly with TLD-MTX as previously published (13). The patient completed the ITI with TLD-MTX safely without any deviation in the protocol.

Around 4 weeks into ERT initiation (one week after completion of TLD-MTX), an upward trend in antibody titers with a peak antibody titer of 25,600 at week 10 was noted. AST (94 U/L) and ALT (66 U/L) were still high at week 10. Due to rising antibody titers, ITI with bortezomib, rituximab, methotrexate, and IVIG was initiated at 13 weeks of ERT as previously published (**Figure 1**) (16). The patient received 2 consecutive cycles (8 doses) of bortezomib with the goal of eliminating antibody-secreting short- and long-lived plasma cells. ITI was discontinued at week 39 on ERT and since then the patient has continued to receive ERT without any additional immunomodulation. ERT dose was increased to 40 mg/kg/week at week 39 on ERT.

**Figure 2:**
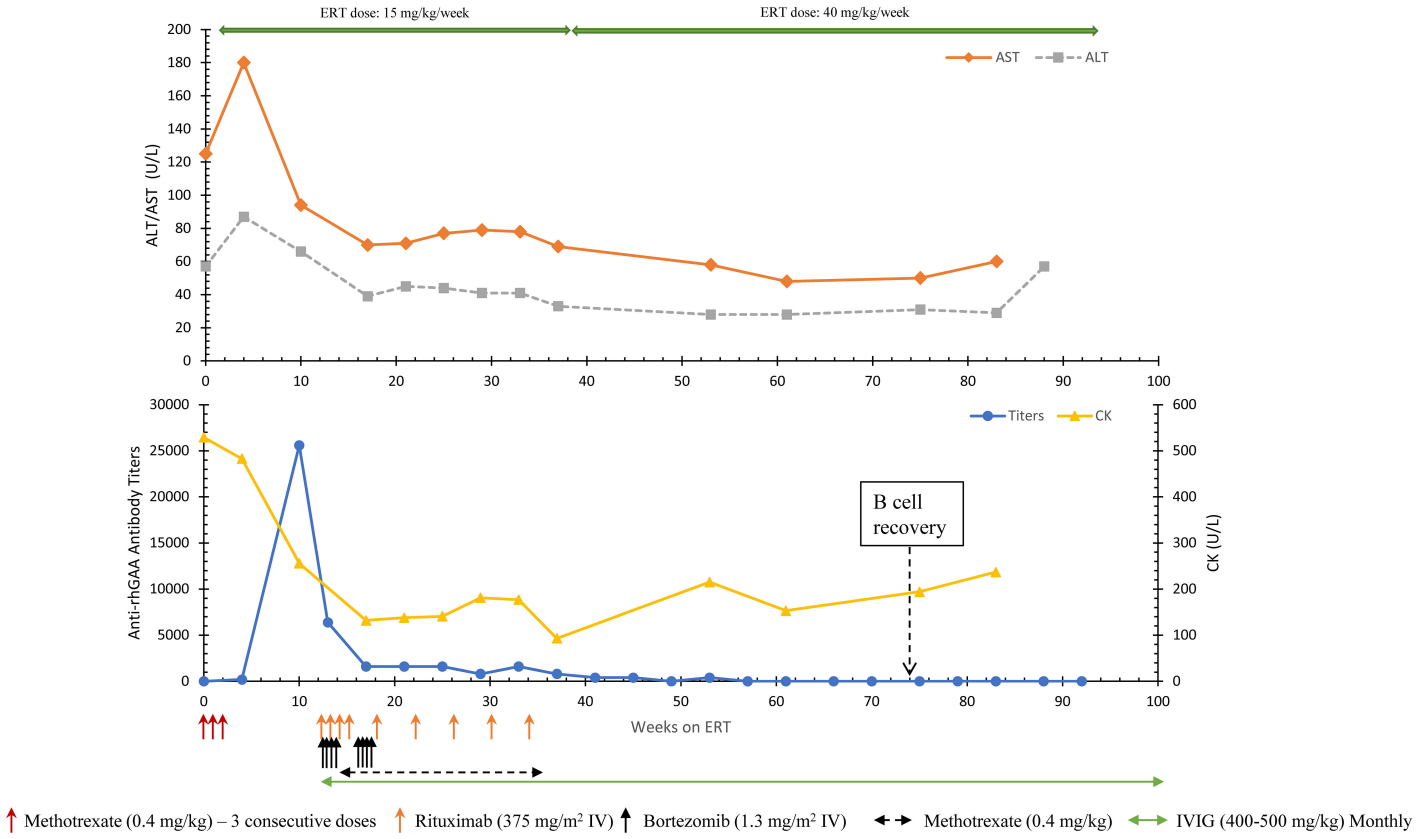
Longitudinal anti-rhGAA antibody titers, AST, ALT, and CK trends in Patient 8. AST, Aspartate aminotransferase; ALT, Alanine aminotransferase; CK, Creatine kinase; ERT, enzyme replacement therapy.

Anti-rhGAA antibody titers decreased from a peak titer of 25,600 (week 10) and remained negative from week 57 until the most recent follow-up (134 weeks) (**Figure 2**). A significant improvement in motor status was noted along with a reduction in antibody titers. At week 19 (4.8 months of age), the patient had delayed motor development (Peabody Developmental Motor Scales-2 (PDMS-2) indicated 3 months age equivalency on the stationery and locomotion subsets) with head-lag, hypotonia, and overall decreased functional abilities. At week 50 on ERT (age 1 year), the patient was performing at an 11-month age equivalency on the stationary subtest, 8-month age equivalency on the locomotion subtest, and 12-month age equivalency on the object manipulation subtest. His gross motor quotient was in the 23rd percentile. At week 45, the patient was feeding orally and did not require respiratory support in comparison to feeding problems observed at the baseline. At week 83, urinary Glc_4_ (4.6 mmol/mol creatinine), CK (237 U/L), and ALT (29 U/L) had normalized; AST (60 U/L) was slightly above the upper limits of normal. The patient received the last dose of rituximab and methotrexate at week 37 and week 39 on ERT, respectively. Complete reconstitution of B cells (i.e., normalization of CD19%) was noted at week 76 on ERT. IVIG was discontinued at week 104 once IgG levels were within normal range. The patient did not have any bacterial infections during immune suppression. At week 34 on ERT, the patient was found to have respiratory syncytial virus (RSV) but did not require hospitalization.

At the follow-up at age 22 months (week 93 on ERT), the patient showed age-appropriate normal development, was feeding by mouth, had a normal LVMI, and normal biomarkers. At week 130 on ERT (age 30.5 months), AST, ALT, and CK were 51 U/L, 24 U/L, and 104 U/L, respectively. No serious adverse events related to ITI were noted. The patient was up to date on the routine vaccinations. The patient has been off all ITI medication, is seronegative for anti-rhGAA (week 134) antibodies, and has been receiving ERT at 40 mg/kg/week since week 22 on ERT (age 5.5 months).

## 4 Discussion

The development of high anti-rhGAA antibody titers can negatively affect the treatment efficacy and clinical outcomes in patients with IOPD. To minimize the negative impact of anti-rhGAA antibodies, immune modulation approaches in the ERT-naïve settings have been utilized with great success and have become a standard of care in patients with IOPD, especially CRIM-negative IOPD. However, a subset of CRIM-positive IOPD patients (32%) can develop significant anti-rhGAA antibodies leading to suboptimal clinical response (20, 23). CRIM-positive IOPD patients who are likely to develop HSAT to ERT show an upward antibody titer trend with titers of ≥12,800 within the first 24 weeks on ERT (20). Published literature has demonstrated that prophylactic ITI is associated with shorter immune suppression and minimal side effects compared to therapeutic ITI. Additionally, patients who have been tolerant to ERT can have a break in tolerance as seen in Patient 5 who developed HSAT after been immune tolerant to ERT for 11 years (18). Considering these, both patients in this report were treated with TLD-MTX ITI in the ERT-naïve setting. Patient 8 completed the protocol without any deviations whereas the ITI administration schedule was altered in Patient 6 due to RSV, viral infections, and severe mucositis. The knowledge gained from an ERT-treated historical cohort of CRIM-positive IOPD patients allowed us to recognize the break in tolerance in these patients, and these data have helped us predict the trajectory of patients destined to develop HSAT (20, 23).

A combination of rituximab, methotrexate, and IVIG has been successful in inducing immune tolerance in ERT-naïve settings, however, this combination had limited success in HSAT patients, most likely due to the presence of short- and long-lived plasma cells. Rituximab targets and effectively depletes cells that express CD20, which includes most members of the B cell lineage from pro-B cells to memory B cells (24). CD19^+^CD20^-^ plasma cells, however, and certain subsets of long-lived memory B cells are resistant to rituximab and have been detected in the weeks to months after treatment for various antibody-mediated diseases (25, 26). Furthermore, B cells with decreased or absent cell-surface CD20 have been frequently detected after rituximab treatment, and are thought to survive as a result of epitope masking, antigen modulation by CD20 internalization, and/or trogocytosis that can allow for B cell escape and persistence [reviewed in (27)]. While treatment of idiopathic thrombocytopenia purpura (ITP) with rituximab to eliminate anti-platelet B cells is initially effective for many (~60%) patients, rituximab treatment itself has also been associated with enhanced differentiation and establishment of long-lived plasma cells in some patients with non-responsive or recurrent disease (25). Data from mouse studies demonstrated that rituximab’s enhancing effect on long-lived plasma cells depends on the influence of splenic CD4^+^ T cells and the cytokine B-cell activating factor (BAFF) (28). Given that some plasma cells and memory B cells can escape depletion by rituximab alone, we recognized the importance of targeting plasma cells by using an agent such as bortezomib during the induction of immune tolerance following the development of HSAT.

In previous studies, we have demonstrated the safety and efficacy of ITI with bortezomib, rituximab, methotrexate, and IVIG in inducing immune tolerance in patients with HSAT (14, 16–19). These include the first known cases where bortezomib was successful in inducing long-term immune tolerance in patients with Pompe disease who developed HSAT. Despite the initial success of the bortezomib-based approach in inducing immune tolerance, we recognized the potential to optimize this approach by identifying patients earlier and using shorter courses of ITI (**Supplementary Figure 2**). The antibody titers in patients 1, 2, 3, and 4 (previously published) peaked at 204,800 to 819,200 and had HSAT for a duration of 22 to 111 weeks before initiation of bortezomib-based ITI (**Table 1**). These cases demonstrated that a delay in intervention and/or lapse in monitoring can result in reduced efficacy of ERT and long-term irreversible tissue damage. All 4 patients (patients 1-4) received a single cycle of bortezomib (4 doses) at the time of ITI initiation.

To achieve tolerance with fewer doses of immunomodulatory drugs, we have focused on improving our management approach for patients with HSAT. We have made it a priority to identify and intervene in these patients as early as possible with the goals of minimizing the impact of anti-rhGAA antibodies on the long-term outcome, shortening the total duration of immunomodulation, and avoiding any serious adverse events from immune suppression. Based on historical data of CRIM-positive IOPD patients, those who ultimately developed HSAT typically seroconverted by a median time of 4 weeks on ERT (range, 4-8 weeks) and had antibody titers of 12,800 by a median time of 10 weeks on ERT (range, 4-24 weeks) (20). The upward trend of antibody titers within the first 24 weeks on ERT stood out as a key feature that differentiated patients with HSAT from those that had persistent low titers; the low titer group did not demonstrate an upward trend in antibody titers after initial exposure to ERT, suggesting their effective immune tolerance to ERT. The data generated from this analysis and the lessons learned from our earlier cases treated with bortezomib-based ITI became the new benchmark that reshaped our approach to immune modulation in patients with HSAT.

In our current approach, a patient with anti-rhGAA antibody titers of 12,800 at two or more time points with or without evidence of a clinical decline/plateau is initiated on a bortezomib-based ITI regimen (29). The first example of our updated approach was presented in a recent case report, in which we reported on a CRIM-negative IOPD patient (patient 7) who was treated with immunomodulation with rituximab, methotrexate, and IVIG with weekly ERT at 40 mg/kg at the age of 4.5 months (**Table 1**) (19). The patient had noticeable initial progress in clinical response to treatment, however, a gradual increase in antibody titers was noticed with antibody titers peaking at 12,800 (week 24). To avoid loss of clinical gains and based on our learning from the historical CRIM-positive IOPD cohort, bortezomib-based ITI (2 consecutive cycles) was initiated without any delay at week 24 which was effective in eliminating anti-rhGAA antibodies (i.e., patient reverted to seronegative). High-dose weekly ERT and timely initiation of ITI with bortezomib allowed the CRIM-negative IOPD patient to achieve normal cardiac function, normal level of skeletal biomarkers, and independent walking at the age of 30 months (19). At the study closure, the patient had been off ITI for 4 months and continued to be seronegative.

The two IOPD patients presented herein, who developed HSAT in spite of their CRIM-positive status, serve as examples of the importance of identifying high-risk CRIM-positive IOPD patients by regular monitoring of anti-rhGAA antibody titers. Both patients received TLD-MTX ITI in an ERT-naïve setting, and while a major deviation in the administration schedule may have contributed to the failure of the initial ITI regimen in patient 6, patient 8 had no such deviation. Furthermore, patient 8 developed antibodies to ERT despite early identification and initiation of ERT within one month of age. As shown in the present study, both patients were initiated on bortezomib-based ITI at the earliest evidence of high titers. A reduction in antibody titers was observed in both cases with antibody titers decreasing from 25,600 to seronegative at the study end. During their initial treatment with bortezomib, patient 8 received 2 consecutive cycles (8 doses) of bortezomib whereas patient 6 received only 1 cycle (4 doses) of bortezomib. The impact of this difference can be seen in the outcome of these two patients; patient 8 required an overall shorter duration of immunomodulation to bring antibody titers down and tolerize to ERT, continued to receive ERT without any ITI, and maintained low antibody titers even after B cell recovery. In contrast, patient 6 required an additional 3 cycles of bortezomib to bring antibody titers down and is still receiving rituximab and subcutaneous IgG. The sum of these experiences (current and previously published cases) strongly suggests that adopting a strategy of early and aggressive intervention at the earliest recognition of high anti-ERT antibody titers may ultimately result in optimized long-term immune tolerance and improved clinical outcomes. Furthermore, the success of a therapeutic regimen meant to eliminate HSAT relies on the inclusion of an agent that effectively targets and eliminates the plasma cells that are responsible for anti-ERT antibodies, such as bortezomib.

Bortezomib is a proteasome inhibitor that was initially used for hematologic malignancies such as multiple myeloma, and targets cells with high rates of protein production by inducing cell death via the terminal unfolded protein response (30). After the discovery that it also targets short- and long-lived plasma cells as a result of their active antibody production (30), it has been found to be efficacious for the treatment of antibody-mediated autoimmune diseases and solid-organ transplant rejection (31). One of bortezomib’s most attractive features in the treatment of antibody-mediated disease is its ability to target non-dividing plasma cells that have high rates of antibody production. Given the ability of CD19^+^CD20^-^ plasma cells to escape death by anti-CD20 targeting agents like rituximab, bortezomib provides a crucial adjunctive therapy to eliminate plasma cells that are generated after ERT therapy is initiated in IOPD. The existence of such cells is demonstrated by the presence of high-titer anti-ERT antibodies. A challenge remains, however, that some memory B cells also appear to be able to survive rituximab therapy by down-regulating CD20, and may therefore be able to re-emerge and participate in recurrent antibody responses (27, 32). In addition to these surviving memory B cells, nascent naïve B cells may participate in recurrent HSAT responses (32). Given that the patients described in this report were able to mount primary HSAT responses despite their CRIM-positive status and their initial ITI, it may suggest that these patients also have an underlying heightened risk for recurrence due to nascent naïve B cells. This further supports our recommendation for close monitoring of such high-risk patients.

From our experience in managing patients with Pompe disease and ITI approaches, we learned that: (1) the dose of bortezomib needed to eliminate the anti-rhGAA antibodies is directly proportional to the duration of anti-rhGAA antibodies, (2) once there is an upward trend of antibody titers, patients do not tolerize by themselves and require some form of intervention to induce tolerance to ERT (20), (3) early intervention with ITI that includes bortezomib allows immune tolerance induction with shorter duration of immune suppression, (4) a minimum of two consecutive cycles of bortezomib is important during the initial round of rescue ITI, and this duration of therapy may therefore be required to optimally target plasma cells, and (5) maintenance rituximab and methotrexate following bortezomib are important to induce long-term tolerance to ERT.

**Figure 3:**
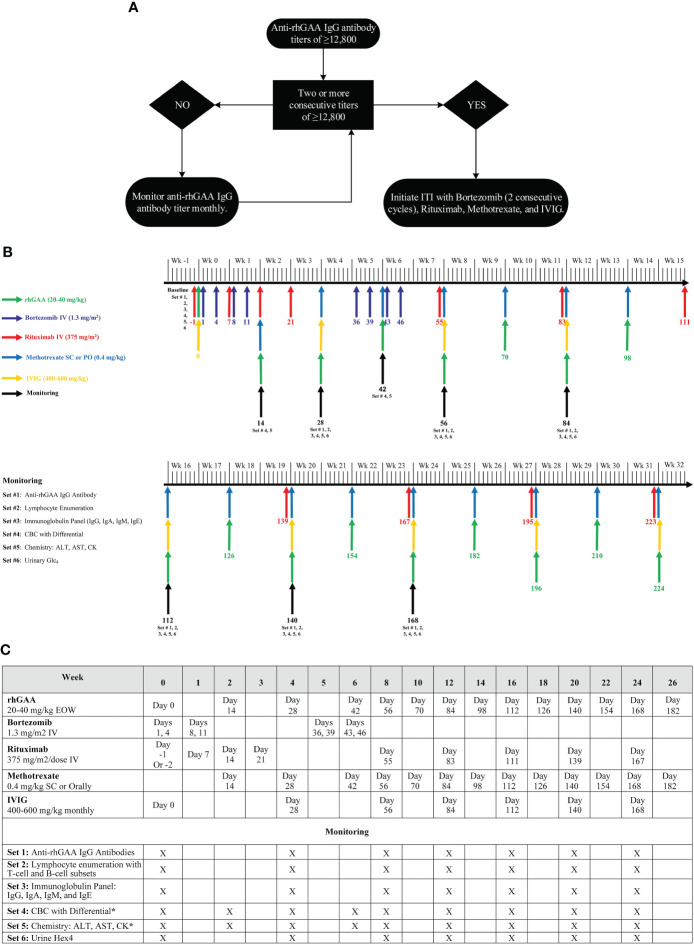
**(A)** An approach for the management of Pompe disease patients with HSAT, **(B)** Bortezomib-based immunomodulation administration and monitoring schedule, **(C)** ITI protocol with bortezomib (2 consecutive cycles), rituximab, methotrexate, and IVIG. **(A)** provides a generalized approach for the management of a Pompe disease patient who developed HSAT (anti-rhGAA IgG antibody titers of ≥12,800). This algorithm provides a framework to determine the timing of a bortezomib-based ITI approach, however, additional details need to be considered before making the final decision. In a patient with a single anti-rhGAA IgG antibody titer of ≥12,800, overall clinical history, previous antibody titers, and biomarkers should be taken into consideration to determine the need for intervention v/s monitoring. Additionally, more importance should be given to the trend of anti-rhGAA IgG antibody titer than a single antibody titer. An upward trend in anti-rhGAA IgG antibody titers, even if the titer is <12,800, warrants monthly monitoring of anti-rhGAA IgG antibody titers to identify if the patient destined to develop HSAT. **(B, C)** Illustrate the timing of immunomodulatory agents’ administration and necessary safety monitoring up to week 32. Beyond week 32, patients should continue to receive maintenance rituximab and methotrexate. Rituximab and methotrexate can be spaced and eventually discontinued depending upon the patient’s anti-rhGAA IgG antibody titers following the initial round of ITI. Some patients may require more than 2 cycles of bortezomib to bring anti-rhGAA IgG antibody titers down. In addition to the safety monitoring described in **(B, C)**, the prescribing information of each agent should be referenced for any additional monitoring. The same ITI administration schedule can be used for Pompe disease patients receiving weekly ERT infusions. Methotrexate daily dose should not exceed 7 mg for a patient weighing <70 kg and should not exceed 10 mg if a patient weighs>70 kg. Withhold vaccination (except Flu shot and RSV-IVIG) while a patient is on treatment with Rituximab and Methotrexate. Immunization with live vaccines can be resumed 9-12 months after the last dose of IVIG (or SCIg). Attenuated vaccines can be resumed right away after complete B cell reconstitution. Titers against vaccines should be performed to assess the adequate humoral response to vaccination and/or the need for reimmunization. rhGAA, recombinant human acid alpha-glucosidase; ITI, immune tolerance induction; CBC, complete blood count; AST, Aspartate aminotransferase; ALT, Alanine aminotransferase; CK, Creatine kinase; Glc_4_, glucose tetrasaccharide; Wk, Week; EOW, every other week; SC, subcutaneous; IV, intravenous. rhGAA applies to any therapeutic protein available as enzyme replacement therapy for Pompe disease including alglucosidase alfa (Lumizyme^®^/Myozyme^®^), avalglucosidase alfa (Nexviazyme^®^), and cipaglucosidase alfa (Pombiliti™). The anti-rhGAA IgG antibody titer cutoff of 12,800 is for alglucosidase alfa and avalglucosidase alfa.

Based on our experience, we have updated our approach to immunomodulation in the setting of an established/entrenched antibody response – (1) a patient with sustained titers of ≥12,800 may benefit from ITI with bortezomib, and (2) ITI is initiated with 2 consecutive cycles of bortezomib (8 doses) with the option to continue for additional cycles based on patient response (**Figure 3**). Further studies are needed to assess the extent of clinical benefits gained by early intervention with ITI in this cohort. In a recently published study, we published the results of multiparameter immunophenotyping and multiplex array analysis elucidating the cellular subsets and cytokine mediators that drive the immune response to ERT in IOPD patients (33). The comparison of low-risk IOPD patients treated with ERT monotherapy with high-risk IOPD patients who received ITI (rituximab, methotrexate, and IVIG) in ERT-naïve, the data suggested that the immune profile of high-risk IOPD patients was similar to low-risk IOPD patients which might be attributed to treatment with ITI. The data suggested that ITI administration changes the immune profile of IOPD patients who were at higher risk of mounting immune response to ERT to more of the immune profile of IOPD patients who have some level of natural tolerance to ERT. This study further demonstrated the success of ITI in inducing immune tolerance, however, identifying these high-risk patients remains a challenge. More importantly, additional methods to identify these high-risk Pompe disease patients at outset are needed; such methods could be incorporated in the Newborn screening algorithm to maximize the benefits of enzyme replacement.

## Data availability statement

The original contributions presented in the study are included in the article/**Supplementary Materials**, further inquiries can be directed to the corresponding author.

## Ethics statement

The studies involving humans were approved by Duke University Health System Institutional Review Board. The studies were conducted in accordance with the local legislation and institutional requirements. Written informed consent for participation in this study was provided by the participants’ legal guardians/next of kin. Written informed consent was obtained from the minor(s)’ legal guardian/next of kin for the publication of any potentially identifiable images or data included in this article.

## Author contributions

AD: Conceptualization, Data curation, Formal analysis, Funding acquisition, Investigation, Methodology, Project administration, Supervision, Visualization, Writing – original draft, Writing – review & editing. GS: Data curation, Formal analysis, Methodology, Writing – original draft, Writing – review & editing. CG: Data curation, Validation, Writing – review & editing. RW: Data curation, Validation, Writing – review & editing. TB: Formal analysis, Methodology, Visualization, Writing – review & editing. PK: Conceptualization, Formal analysis, Funding acquisition, Investigation, Methodology, Project administration, Resources, Supervision, Writing – original draft, Writing – review & editing.

## Supplementary material

Supplementary Figure 1
**(A, B)** Longitudinal immunoglobulin levels in patients 6 and 8. IgG, immunoglobulin G; IgA, immunoglobulin A; IgM, immunoglobulin M; IgE, immunoglobulin E; ERT, enzyme replacement therapy.

Supplementary Figure 2 Timeline of evolved bortezomib-based ITI. Patients 1-4 represent the first cases of patients who developed high-sustained anti-rhGAA IgG antibody titers and were successfully immune tolerized with bortezomib-based ITI. In these patients, ITI was initiated after the development of extremely high anti-rhGAA IgG antibody titers (≥204,800). Patients 5 and 6 represent the next iteration in our approach in whom bortezomib-based ITI was initiated relatively early before developing extremely high anti-rhGAA IgG antibody titers, however, only single-cycle of bortezomib was used at the time of initiation and additional cycles were added based on the response to the initial first cycle of bortezomib. Patients 7 and 8 represent our current approach of early intervention with a minimum of 2-cycles of bortezomib. ITI, immune tolerance induction.
