# Supplementary figures and images for: An updated management approach of Pompe disease patients with high-sustained anti-rhGAA IgG antibody titers: experience with bortezomib-based immunomodulation
Source: Front Immunol. 2024 Mar 8;15:1360369. doi: 10.3389/fimmu.2024.1360369 (PMC10959098; doi:10.3389/fimmu.2024.1360369)

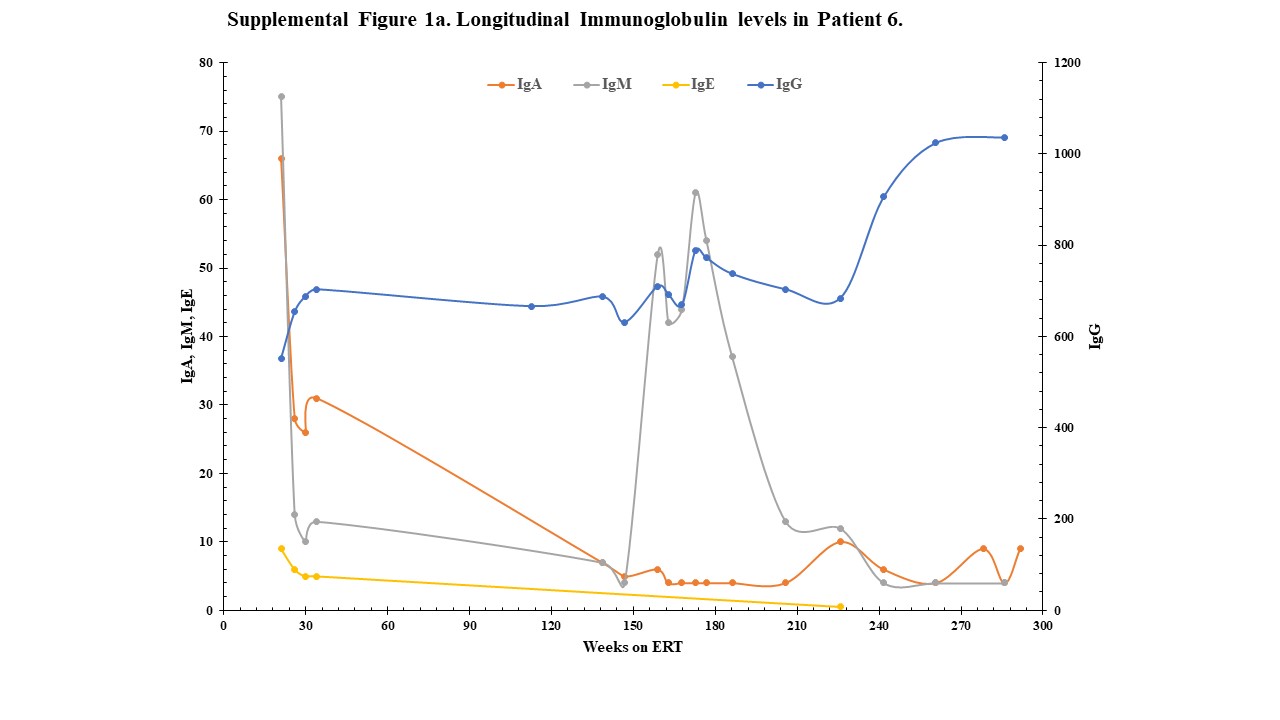

Supplement: Supplementary Figure 1 — (A, B) Longitudinal immunoglobulin levels in patients 6 and 8. IgG, immunoglobulin G; IgA, immunoglobulin A; IgM, immunoglobulin M; IgE, immunoglobulin E; ERT, enzyme replacement therapy. [file Image_1.jpeg]

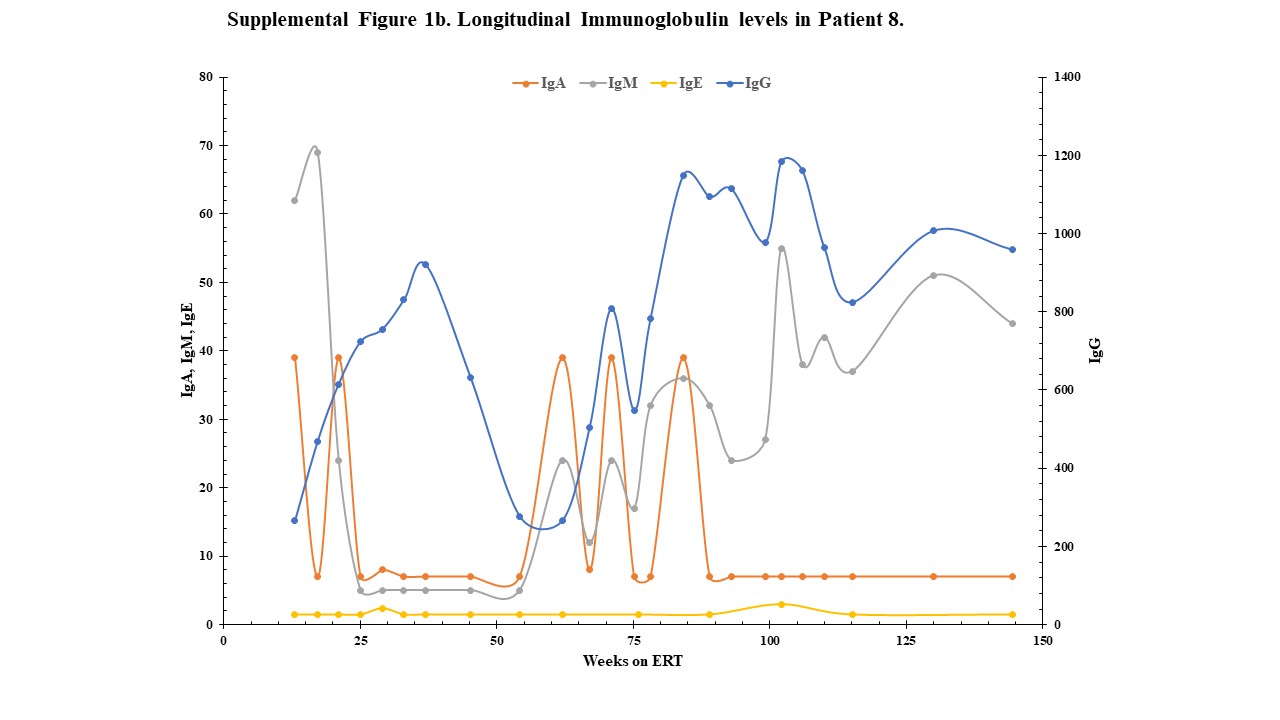

Supplement: Supplementary Figure 2 — Timeline of evolved bortezomib-based ITI. Patients 1-4 represent the first cases of patients who developed high-sustained anti-rhGAA IgG antibody titers and were successfully immune tolerized with bortezomib-based ITI. In these patients, ITI was initiated after the development of extremely high anti-rhGAA IgG antibody titers (≥204,800). Patients 5 and 6 represent the next iteration in our approach in whom bortezomib-based ITI was initiated relatively early before developing extremely high anti-rhGAA IgG antibody titers, however, only single-cycle of bortezomib was used at the time of initiation and additional cycles were added based on the response to the initial first cycle of bortezomib. Patients 7 and 8 represent our current approach of early intervention with a minimum of 2-cycles of bortezomib. ITI, immune tolerance induction. [file Image_2.jpeg]

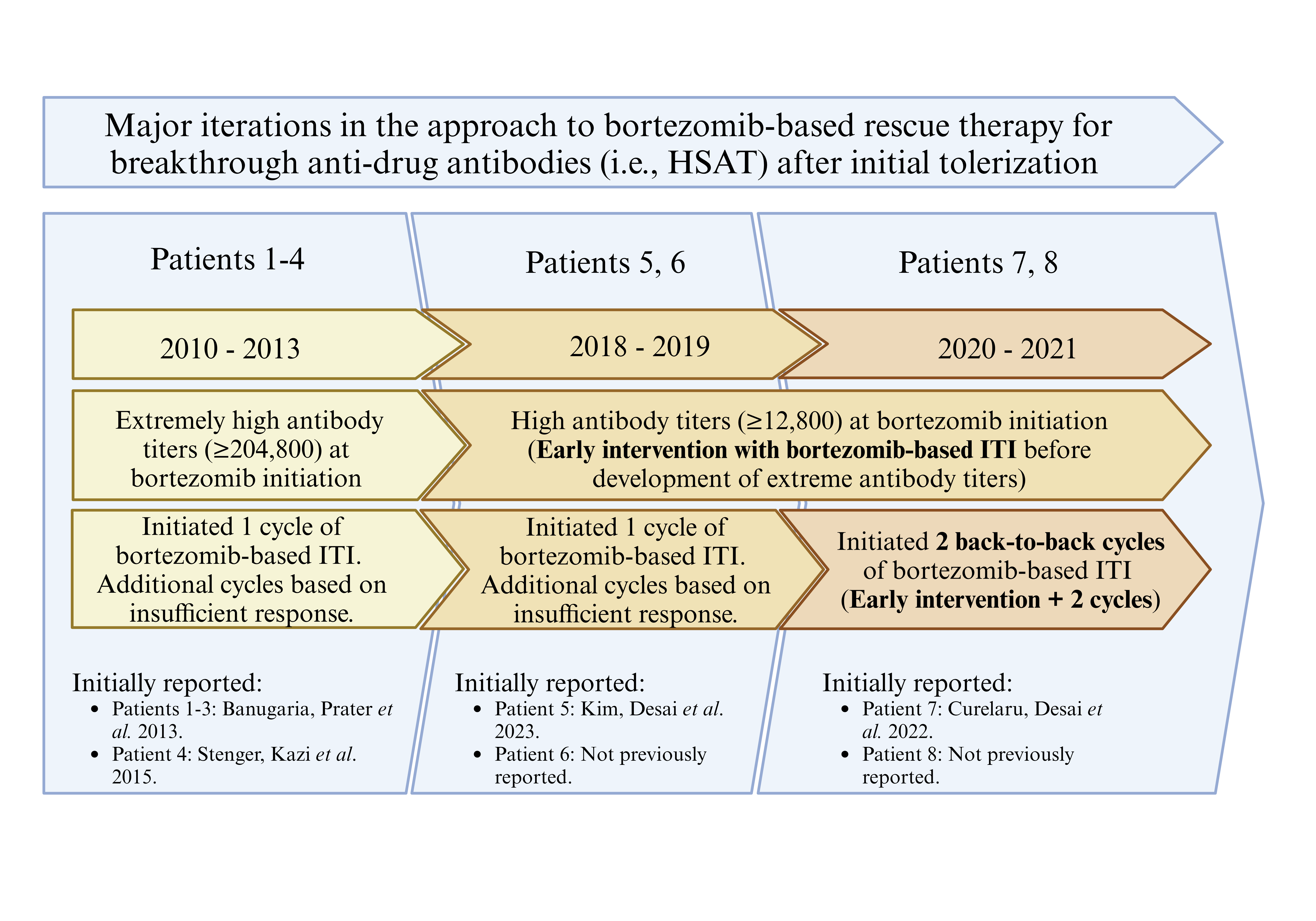

Supplement: Supplementary file 3 [file Image_3.png]
